# Mir-16 Decreases the Expression of VTI1B and SMPD1, Genes Involved in Membrane-Protein Trafficking in Melanoma

**DOI:** 10.3390/cancers17132197

**Published:** 2025-06-29

**Authors:** Adi Layani, Tal Meningher, Yechezkel Sidi, Dror Avni, Raya Leibowitz

**Affiliations:** 1 Laboratory of Molecular Cell Biology, Department of Medicine C, Sheba Medical Center, Tel Hashomer, Ramat Gan 52621, Israel; 2 Gray Faculty of Medical and Health Sciences, Tel-Aviv University, Tel-Aviv 69978, Israel; 3 Oncology Institute, Shamir Medical Center, Be’er Ya’akov 70300, Israel

**Keywords:** melanoma, miR-16, miR-15/16 family, checkpoint proteins, autophagy, SMPD1, VTI1B

## Abstract

**Introduction:** The interface between T cells and the tumor microenvironment, termed the ‘immunological synapse’, consists of multiple checkpoint protein pairs co-expressed on both sides of the synapse. mir-16, a microRNA from a widely known tumor-suppressor family of miRNAs, was previously shown by us to be downregulated in melanoma. As other miRNAs from this family have been shown to directly target checkpoint proteins, here we investigated whether miR-16 influences the expression patterns of checkpoint proteins in melanoma. **Methods:** Single-cell gene expression data from the melanoma microenvironment were retrieved from a public database. Melanoma cell lines were established from metastatic lesions and transiently transfected with an hsa-miR-16-5p-mimic RNA or a mir-16-expressing plasmid. The mRNA expression profiles were analyzed using an Affymetrix microarray. Direct targets of miR-16 were identified by luciferase reporter assays. Protein levels were assessed by Western blotting. **Results:** Bioinformatic analysis revealed that the expression levels of eight checkpoint mRNAs, known to be present on the melanoma side of the immunological synapse, were highly correlated. Four of these mRNAs contained putative binding sites for the miR-15/16 family. miR-16 expression was significantly reduced in melanoma cells, compared to normal melanocytes. Luciferase reporter assays demonstrated that miR-16 directly targets the 3′ untranslated regions (3′UTRs) of CD40, CD80. The mRNAs downregulated following miR-16 overexpression were highly enriched for genes involved in autophagy, vesicle-mediated transport, and the regulation of protein membrane localization. Among these, VTI1B and SMPD1 were confirmed to be direct targets of miR-16. Transient overexpression of miR-16 resulted in a significant reduction in SMPD1 and VTI1B levels in melanoma cell lines. **Conclusions:** Our findings suggest that miR-16 potentially modulates melanoma tumorigenesis, metastasis and immunogenicity by altering the composition of checkpoint proteins at the immunological synapse and by regulating cellular pathways associated with intracellular trafficking and transmembrane protein presentation.

## Simple Summary

Melanoma cells, like many cancer cells, can evade the immune system by displaying special proteins on their surfaces called ‘immune checkpoints’. Drugs known as ‘checkpoint inhibitors’ target these proteins and have transformed melanoma treatment, but not all patients benefit from this treatment. In our previous study, we found that the expression of miR-16—a known ‘tumor-suppressor micro-RNA’—is significantly reduced in melanoma compared to normal human epidermal melanocytes. In this study, we investigated whether there is a correlation between miR-16 and immune checkpoint expression in melanoma. We found that miR-16 potentially has such an effect on two levels. First, it can directly regulate two checkpoint genes, CD40 and CD80. Second, miR-16 regulates the expression of VTI1B and SMPD1, which are important proteins for transporting, clustering, and modulating proteins on cell membranes. Through this second effect, miR-16 may affect the expression of checkpoint proteins in general. Our findings offer new potential insights on how checkpoint proteins are regulated in melanoma and warrant further research. This knowledge could one day help make melanoma cells less able to evade the immune system and more responsive to immunotherapy treatments.

## 1. Introduction

Melanoma, a potentially fatal skin cancer originating from melanocytes at the dermal–epidermal junction, remains a significant health burden and is among the solid cancers with rising incidence [1]. The advent of immune checkpoint inhibitors has transformed the treatment landscape for melanoma; however, not all patients achieve an effective response, and some may experience disease progression despite initial therapeutic benefits [2], highlighting the urgent need to further melanoma research and to find additional therapeutic avenues. Currently approved therapies primarily target key immune checkpoints, including CTLA-4, a co-inhibitory protein on T lymphocytes that interacts with B7.1 (CD80) and B7.2 (CD86) on antigen-presenting cells; PD-1, another co-inhibitory protein on T lymphocytes that engages with PD-L1 within the tumor microenvironment; PD-L1 itself; and more recently, LAG-3, which interacts with major histocompatibility complex (MHC) proteins [3]. Despite these advancements, the interaction between T cells and the tumor microenvironment, often referred to as ‘the immunological synapse’ (IS), involves numerous additional checkpoint proteins and the vital T-cell receptor (TCR)–MHC pairing. The immunological synapse is not a classical synapse involving the transport of small protein transmitters across a physical cleft between adjacent cells; rather, it represents a conceptual framework of cell–cell interactions, in which the collective effect of the proteins expressed on both membranes determines the signal received by each cell. Investigating the factors that influence the expression and localization of checkpoint proteins, MHC, and TCR at the IS may illuminate strategies for enhancing the immunogenicity of melanoma and improve its responsiveness to checkpoint inhibitors.

While it is well-established that checkpoint proteins are co-expressed on T cells [4], they are also co-expressed on melanoma cells within the immunological synapse [5]. CD40 has long been shown to be expressed in melanoma ([6,7] and later [8]). PD-L1 and PD-L2 were also shown to be expressed by melanoma cells, albeit with significant heterogeneity, and to be associated with the extent of T-cell infiltration [9]. In contrast, B7-1 (CD80) is not constitutively expressed in melanoma cells, but its ectopic expression increases melanoma immunogenicity [10].

Micro-RNAs (miRNAs) serve as ‘master regulators’ of gene expression and have long been implicated in various cancer-related mechanisms [11]. The micro-RNA ‘family’ mir-15/16, containing several miRNAs with the same seed sequence, was the first identified ‘tumor-suppressor’ family in chronic lymphocytic leukemia [12]. Serum levels of mir-16 were found to be lower in melanoma patients than in healthy subjects in a hospital-based case-control study [13]. Subsequent work directly proved the tumor-suppressing effects of mir-16 in melanoma, and delved into its molecular mechanism, demonstrating that it directly binds to SOX10 and regulates its expression. This work also demonstrated that ETS1, an important mediator of epithelial–mesenchymal transition, directly bound to the promotor of the mir-16 gene and inhibited its expression [14]. A recent review showed that mir-16 is indeed tumor-suppressive and that its down-regulation characterizes many types of solid cancer [15].

Notably, miR-424, a member of the miR-15/16 family, has been shown to directly target the 3′ untranslated region (UTR) of both CD80 and PD-L1 in an ovarian cancer model [16]. This observation prompted the question of whether mir-16 from this family also influences the expression and localization patterns of checkpoint proteins in melanoma.

## 2. Materials and Methods

### 2.1. Single Cell Expression and Tumor Cancer Genome Atlas (TCGA) Data Analysis

Gene expression data of single melanoma and non-malignant T cells, B cells, NK cells, CAFs, endothelial cells, and macrophages were obtained from the Gene Expression Omnibus database (GSE72056, GSE77940) [17]. TCGA gene expression data from RNA sequencing were downloaded from public TCGA repositories.

### 2.2. Generation of Melanoma Cell Lines and Transfection

Melanoma cell lines (14PA, mel333A1) were generated directly from metastatic melanoma lesions of patients at the surgical branch of the NIH or the ‘Ella institute for melanoma research’ at the Sheba Medical Center and have been previously extensively investigated by us [18]. The melanoma cell line HS294t was obtained from the American Type Culture Collection (ATCC, Manassas, VA, USA). The cell lines were grown in DMEM medium supplemented with 10% fetal bovine serum (FBS), 1% penicillin–streptomycin antibiotics, 1%L-glutamine and 2.5% HEPES solution (Biological Industries, Kibbutz Beit Haemek, Israel). Melanoma cell lines were transiently transfected with an hsa-miR-16-5p-mimic RNA (Assay ID MC10339, Thermo Fisher Scientific Inc., Waltham, MA, USA) using Lipofectamine™ RNAiMAX Transfection Reagent (Thermo Fisher Scientific Inc.).

### 2.3. Cloning

A miR-16 expressing plasmid was created by annealing miR-16-pre-miRNA oligonucleotide primers (following the addition of nucleotides at their edges to generate HindIII and EcoRI cleaved sites) and cloning them into the HindIII + EcoRI cut pcDNA3.1 (+) plasmid.

The 3′UTR region of either SMPD1, VTI1b, CD80, or CD40 were PCR-amplified from human genomic DNA with MyTaq HS Red Mix (Bioline Reagents Ltd., London, UK) using the primers shown in Table 1. The amplified DNA was extracted from agar gel using a Zymoclean Gel DNA Recovery Kit (Zymo Research, Irvine, CA, USA). The fragment was cloned into the psiCHECK™-2 Vector (Promega Corporation, Madison, WA, USA) and cleaved with NotI and XhoI (New England Biolabs, Ipswich, MA, USA) using In-Fusion^®^ Snap Assembly Master Mix (Takara Bio, San Jose, CA, USA).

### 2.4. RNA Extraction and Analysis

Total RNA was extracted from the cell lines by using a Norgen Total RNA Purification Kit (Norgen, Thorold, ON, Canada). Quantity and quality were evaluated using a Nanodrop ND-2000 (Thermo Fisher Scientific Inc.) with inclusion criteria of A260/A280  ≥  1.8. The mRNA expression profiling was performed using Affymetrix PrimeView oligonucleotide arrays according to the manufacturer’s protocol and as previously described by us at length [18].The probe sets contained in the arrays were analyzed using an RMA algorithm. Hierarchical clustering was performed using the Spotfire DecisionSite for Functional Genomics (Somerville, MA, USA).

### 2.5. Luciferase Reporter Assays

The protocol was detailed at length in our previous publication [18]. A total of 500,000 cells of HEK293 were seeded in 24-well tissue culture dishes. Then, 24 h later, cells were transfected with 50 ng of a reporter plasmid or mimic miR-16 RNA, using LipofectamineTM 2000 Reagent (Invitrogen-Thermo Fisher Scientific) according to the manufacturer’s protocol. The ‘Dual-Luciferase Reporter (DLR) Assay System’ (Promega) was applied 24 h after transfection according to the manufacturer’s protocol. Data is represented as mean ± SEM from 3 independent experiments.

### 2.6. Western Blot Analysis

Whole cell lysates were prepared in cell lysis buffer (50 mM Tris-Hcl, 150 mM NaCl, 0.5% sodium deoxycholate, 0.1% SDS, 1% NP-40) and supernatants collected by centrifugation. The protein concentration was measured using the Bradford assay (Bio-Rad, Hercules, CA, USA), and 30 μg of each protein sample were denatured in SDS sample buffer and separated on 10–15% SDS–PAGE. Separated proteins were transferred to nitrocellulose membranes and the blots were blocked using 5% skim milk in PBST. Proteins were reacted with the following antibodies: Anti-Human SMPD1 (R&D Systems, Minneapolis, MI, USA, Catalog #: MAB5348) diluted 1:1000; Anti-Human VTI1b (BD Transduction Laboratories, Franklin Lakes, NJ, USA, Catalog No:611404); anti-GAPDH (Cell Signaling Technology, Danvers, MA, USA, Cat. No. 2118), Goat anti-hamster-HRP IgG (Jackson Immuno-Research Lab, West Grove, PA, USA) or Goat anti-mouse IgG (Sigma-Aldrich, Jerusalem, Israel), both diluted 1:10,000; enhanced chemiluminescence (ECL) was performed with WESTAR ANTARES (Cyanagen, Bologna, Italy). Quantitation was performed with the Image Lab program (Bio-Rad Laboratories).

### 2.7. Statistical Analysis

Spearman rho coefficients were calculated for association and Student’s *T*-test was performed to compare two groups of variables. When multiple comparisons were performed, the *p* value was corrected using the False-Detection-Rate (FDR) method [19]. Statistical tests and graphing were performed using GraphPad Prism version 5.01, August 2007, for Windows (GraphPad Software; www.graphpad.com, accessed on 20 August 2024).

## 3. Results

### 3.1. Immune Checkpoint mRNAs Are Co-Expressed in the Melanoma Microenvironment

**Figure 1:**
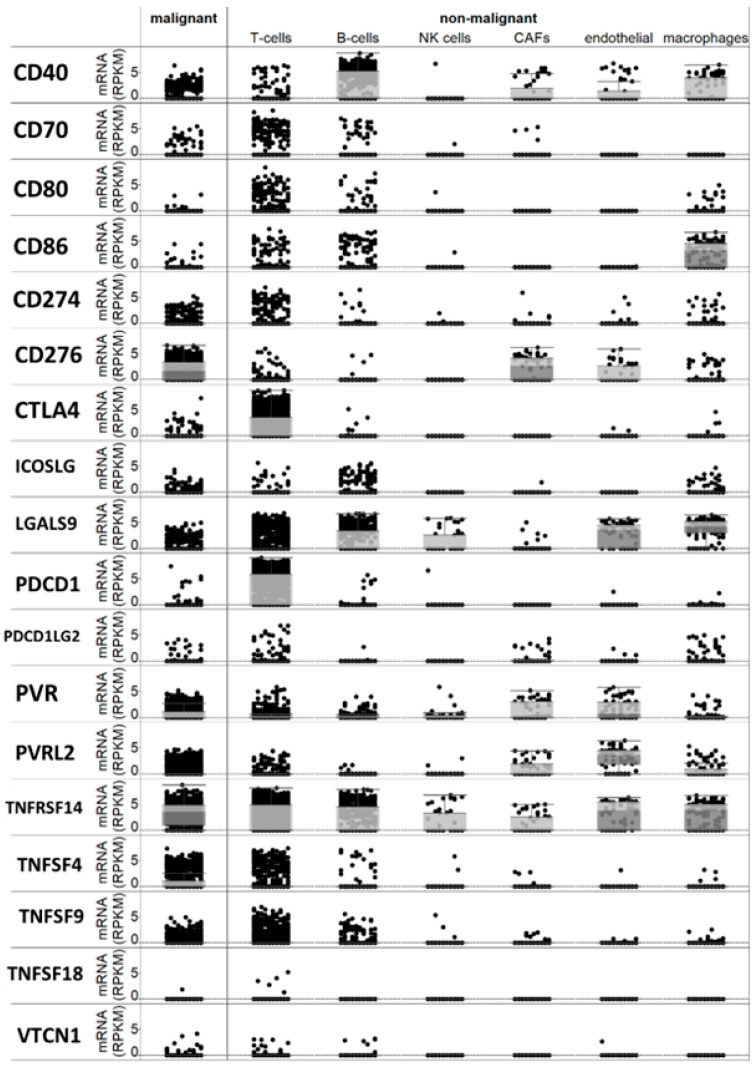
Expression of checkpoint mRNAs in single melanoma cells and in several types of immune and micro-environment cells.

Following a meticulous review of the available literature, we compiled a list of over twenty checkpoint genes that had been suggested to be expressed in melanoma or antigen-presenting cells and implicated in immune regulation within the tumor microenvironment. A bioinformatics analysis of a single-cell RNA sequencing database [17] revealed that the majority of these genes are expressed, at least to some extent, in melanoma cells or non-immune cells within the tumor microenvironment (Figure 1). Specifically, PD-L1 (CD274) mRNA was detected in melanoma cells, along with CD40 and B7-H3 (CD276) mRNAs. Additionally, B7-H2 (CD86) mRNA was found in macrophages, PDCD1 (PD-1) mRNA was present in T cells (as expected), and TNFRSF14 and TNFSF9 mRNAs were observed in both T and melanoma cells (Figure 1).

Analysis of the TCGA database demonstrated that the expression of eight of these mRNAs was significantly correlated with one another, yielding Spearman correlation coefficients greater than 0.5 (corrected q-value < 0.1; Table 2). This subset included the co-stimulatory checkpoints TNFSF4, ICOSLG, and CD40, the co-inhibitory checkpoints PD-L1 (CD274), PD-L2 (PDCD1LG2), and Galectin-9 (LGALS9), as well as the context-dependent checkpoints B7.1 (CD80) and B7.2 (CD86). 

### 3.2. Several Co-Expressed Checkpoint Genes Contain Putative Binding Sites for miRNAs from the miR-15/16 Family

**Figure 2:**
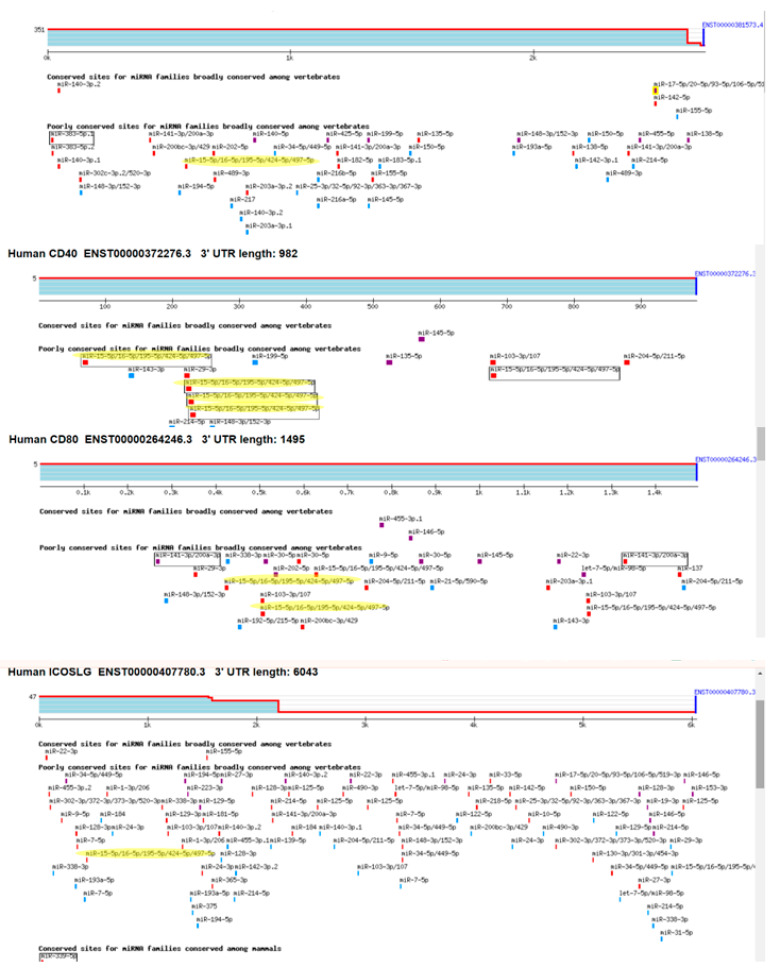
Putative binding sites for mir-15/16 miRNA in the 3′UTR of 4 co-expressed checkpoint mRNAs–CD274 (PD-L1), CD40, CD80, and ICOSLG.

The observed network of co-expressed mRNAs suggests potential joint regulation by micro-RNAs acting as ‘master co-regulators.’ Utilizing the publicly available software ‘TargetScan’ (version 7.2) [20], we determined that four (50%) of the highly co-expressed mRNAs contained putative binding sites for the miR-15/16 family of miRNAs within their 3′ UTR regions: PD-L1 and ICOSLG (one binding site each), CD80 (two binding sites), and CD40 (four binding sites) (Figure 2). The miR-15/16 family of miRNAs, all sharing a common seed sequence, has been implicated in various cancers through multiple mechanisms, including modulation of checkpoint genes [21]. Hence, we sought to further investigate the relationship between miRNAs from the miR-15/16 family and checkpoint gene expression in melanoma.

### 3.3. miR-16 Over-Expression Leads to Reduced Expression of CD40 and CD80 mRNAs

**Figure 3:**
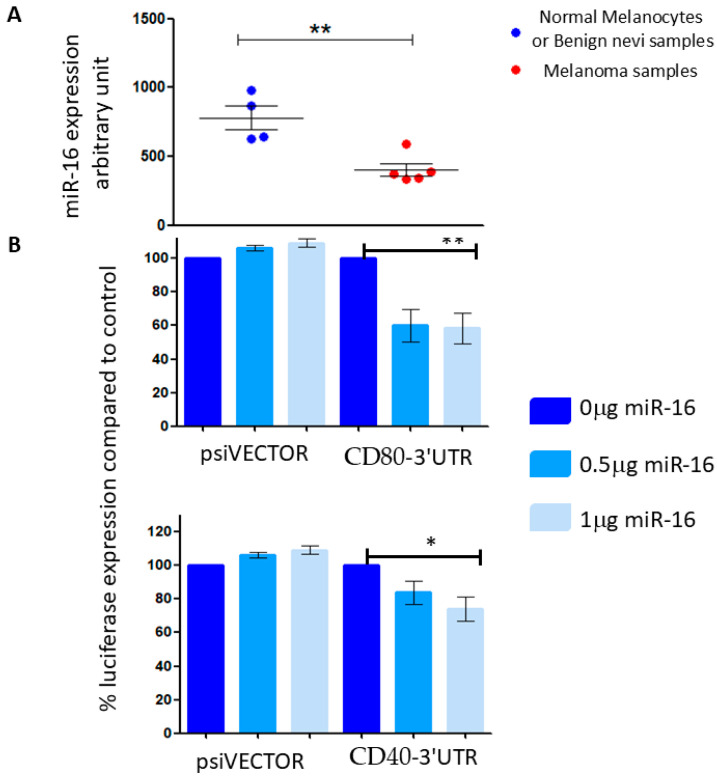
(**A**) Mir-16 expression in normal human epidermal melanocytes and benign nevi (blue dots) and melanoma cell lines (red dots), from a miRNA array. (**B**) 293T cells were co-transfected with a vector containing a part of the 3′UTR of CD80 (left) or CD40 (right) concomitant with a miR-16-expressing vector at different concentrations (0.5 or 1 μg). The results are presented as the ratio of expression of renilla/luciferase that was normalized relative to control-transfected cells for each vector. Data is represented as mean ± SEM from 3 independent experiments. * *p* < 0.05, ** *p* < 0.01.

Data from our previously conducted miRNA arrays (published initially in [22]) indicated that miR-16 expression is significantly reduced in melanoma, compared to its expression in normal human epidermal melanocytes (Figure 3A), reinforcing the proposed role of miR-16 as a tumor suppressor in melanoma [13,14]. Subsequently, we focused our research efforts on this miRNA. Reporter assays demonstrated that overexpression of miR-16 resulted in a 15–25% reduction in luciferase expression when linked to the 3′ UTRs of CD40 and CD80 (Figure 3B). These findings suggest that the 3′ UTRs of CD40 and CD80 are direct targets of miR-16.

### 3.4. Over-Expression of miR-16 Leads to a Significant Decrease in the Expression of mRNAs Involved in Intracellular Vesicle-Mediated Transport and Protein Trafficking

**Figure 4:**
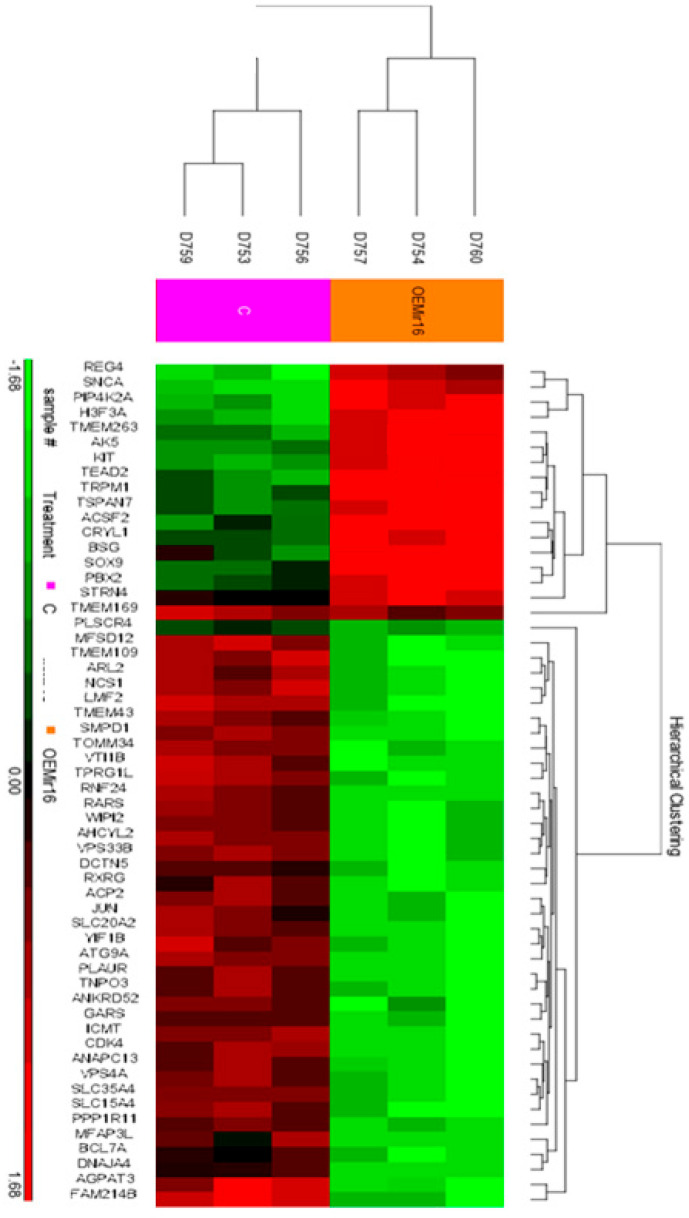
Heat-map of hierarchical clustering analysis based on significantly changed mRNAs. Each row represents an mRNA, and each column represents a sample. The sample clustering tree is shown at the top. The color scale shown in the map illustrates the relative expression levels of mRNAs across samples: red represents high expression levels, and green represents lower expression levels.

To rigorously investigate the effects of miR-16 expression on mRNA levels in melanoma, we overexpressed miR-16 in 14PA melanoma cells and performed mRNA arrays using the Affymetrix expression array platform. This analysis identified a significant downregulation of 28 genes, each decreasing by at least 1.3-fold (Figure 4).

Bioinformatics analysis revealed that the majority of these genes are potential targets of miRNAs from the miR-15/16 family; however, this list did not include the aforementioned checkpoint genes.

We subsequently conducted gene ontology classification analysis on the downregulated genes (depidcted in Table 3) using the PANTHER Functional Annotation Tool [23,24,25]. This analysis revealed significant enrichment for genes involved in autophagy (*p*-value = 0.00073), regulation of protein localization to the plasma membrane (*p*-value = 0.013), and vesicle-mediated transport (*p*-value = 0.025; Table 4).

### 3.5. miR-16 Directly Targets the 3′UTR of SMPD1 and VTI1B and Decreases Their Protein Expression

**Figure 5:**
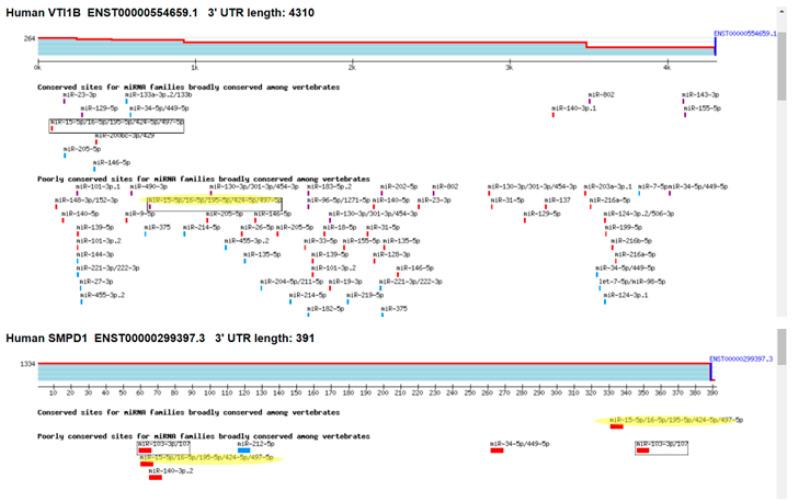
Putative binding sites for miR-15/16 miRNA in the 3′UTR of VTI1B and ASM (SMPD1). The yellow marking represents the putatin bindimg sites for mir-15/16 miRNAs. The different color bars represent differing binding of miRNA seed sequences.

Among the identified genes, VTI1B exhibited a 1.6-fold decrease in expression in cells overexpressing miR-16, and it contains one putative binding site for miRNAs from the miR-15/16 family in its 3′ UTR. The gene with the most substantial decrease in expression was SMPD1 (ASM), which decreased by 2.5-fold and contains two binding sites for miR-15/16 miRNAs (Figure 5).

**Figure 6:**
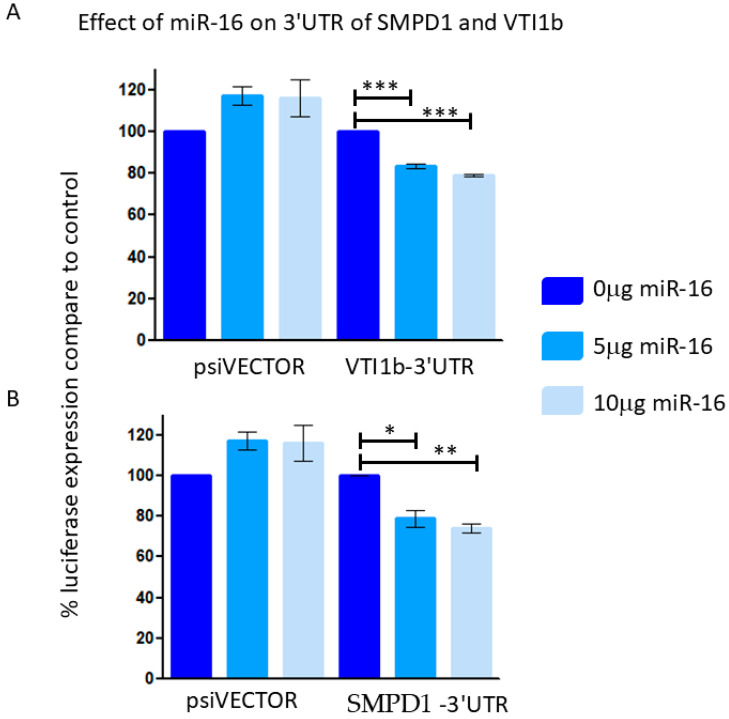
293T cells were co-transfected with control empty vector (vector) or with a vector containing a part of the 3′UTR of VTI1B (**A**) or SMPD1 (**B**), concomitant with a miR-16 expressing vector at different concentrations (0.5 or 1 μg). The results are presented as the ratio of expression of renilla/luciferase that was normalized relative to control-transfected cells for each vector. Data is represented as mean ± SEM from 3 independent experiments. Repeated Measures ANOVA; Tukey’s Multiple Comparison Test. * *p* < 0.05, ** *p* < 0.01, *** *p* < 0.001.

Reporter assays indicated that miR-16 led to an approximately 20% reduction in luciferase expression when linked to the 3′ UTRs of VTI1B and SMPD1 (Figure 6), suggesting that these 3′UTR are direct targets of miR-16.

**Figure 7:**
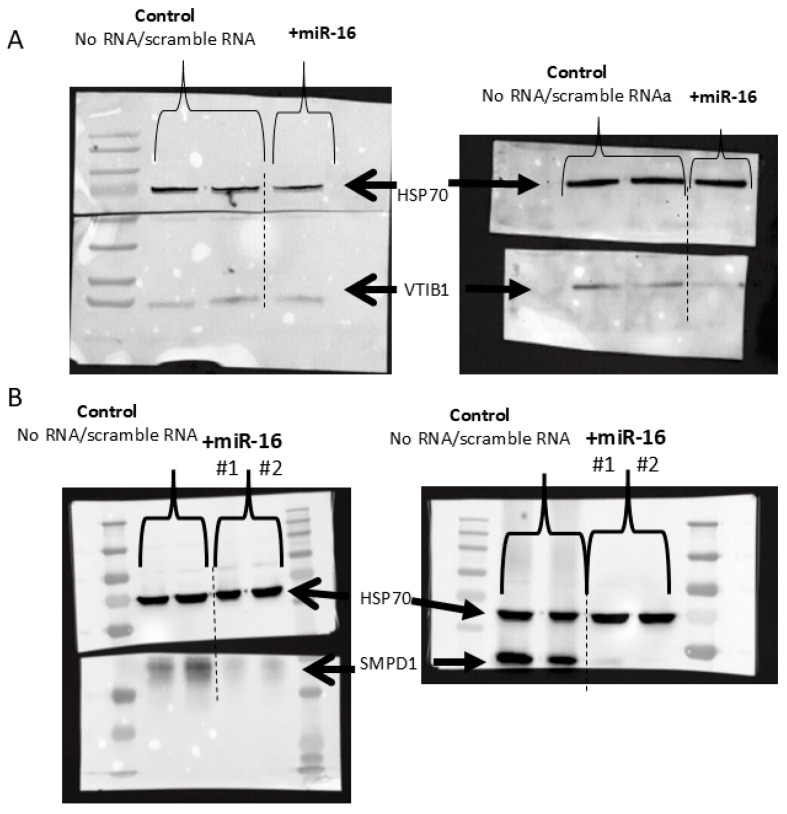
Western blot analysis of VTI1B (**A**) and SMPD1 (**B**) following transfection of two melanoma cell lines with scramble or miR-16-mimic RNA. HSP70 served as an internal loading control.

Last, we analyzed the protein level of SMPD1 (ASM) and VTI1B in two melanoma cell lines overexpressing miR-16, using Western blot analysis. Transfection with mimic miR-16 RNA led to significant decreases in both SMPD1 and VTI1B (Figure 7).

## 4. Discussion

This study was prompted by our observation that several checkpoint mRNAs expressed on the cancer/APC side of the immunological synapse exhibited strong co-expression patterns in the TCGA melanoma dataset. We hypothesized that a microRNA, such as miR-16 from the miR-15/16 family, might act as a master regulator of this expression profile.

### 4.1. Direct Effects of miR-16 on Checkpoint Proteins

Our results show that miR-16 directly targets the 3′UTRs of CD40 and CD80, two important modulators of the tumor immune microenvironment. While novel for miR-16, this regulatory mechanism is supported by previous findings demonstrating the targeting of similar checkpoints by miR-424 and miR-503 [16,26]. Of note, the effect of mir-424/503 [26] has been found to be stronger than our observed effect. This may indicate that additional sequences outside the seed-sequence alter the targeting potential of miRNAs; indeed, this is a known phenomenon. These observations suggest that the miR-15/16 family broadly influences checkpoint protein composition at the tumor immunological synapse. That which affects the specific targeting of miRNAs relative to their cognate mRNAs remains to be studied.

### 4.2. Direct Effects of miR-16 on VTI1B and SMPD1

Interestingly, overexpression of miR-16 did not significantly downregulate checkpoint mRNAs in melanoma cell lines, highlighting potential limitations of in vitro systems. However, we observed a consistent downregulation of genes involved in intracellular vesicle-mediated transport, protein trafficking, and autophagy, including YIF1B, ATG9A, VTI1B, VPS33B, and VPS4A.

We identified SMPD1 (ASM) and VTI1B as direct targets of miR-16. Both proteins are critical for intracellular trafficking pathways: SMPD1 facilitates ceramide production essential for receptor clustering, including CD40 and CD95 [27,28], and regulates trafficking of palmitoylated proteins like PD-L1 [29]. Downregulation of SMPD1 has been associated with reduced tumor growth, impaired metastasis, and altered Met receptor trafficking [30], suggesting that miR-16 may suppress melanoma progression by disrupting these pathways.

SMPD1 is an enzyme that hydrolyses sphingomyelin to ceramide. This is a critical step in the activation of several receptors. Sphingolipid- and cholesterol-enriched membranes form domains named rafts [31]. These lipid rafts harbor many receptors and regulatory molecules [32]. Because of the sphingolipid and cholesterol enrichment in these rafts, these structures and the proteins harbored within them are separated from other phospholipids in the cell membrane. Upon SMPD1 activation, sphingomyelin is hydrolyzed to ceramide. This dramatically changes the biophysical properties of rafts. Ceramide-enriched membranes spontaneously self-associate and have the tendency to form small microdomains which are able to fuse to one or a few large ceramide-enriched microdomains to form platforms. These ceramide platforms play a role in the re-organization of membrane receptors, including their clustering, which is essential for the activation of these receptors [31,32]. It is tempting to speculate that by regulating the expression of SMPD1, mir-16 may affect the cellular ability to cluster receptors and to respond to outside signaling. Clearly this needs to be demonstrated in future studies.

VTI1B is involved in cell membrane trafficking, specifically in the Golgi apparatus [33]. It was previously shown to co-precipitate with Synaxtin-7, which was highly expressed in a murine melanoma cell line [34], but the functional significance of this complex has not been elucidated. Upon B-cell receptor activation, VTI1b co-localizes with the antigen–B-cell-receptor complexes in the immune synapse [35]. This may also suggest that miR-16 affects the structure and function of the immune synapse on the cell surface by inhibiting VTI1B.

While inhibition of autophagy or SMPD1 (through downregulation by miR-16 or additional mechanisms) could enhance tumor immune evasion by reducing antigen presentation and increasing PD-L1 stability [36,37], the broader tumor-suppressive effects of miR-16, including its documented targeting of oncogenes like SOX4 and TP53 [14], and its reduced expression in melanoma patients [13], emphasize its multifaceted role in cancer control.

## 5. Conclusions

The miR-16, a well-known tumor suppressor miRNA already implicated in past work in melanoma, may potentially exert its known tumor-modulating effects in melanoma by altering checkpoint expression at the immunological synapse and by altering intracellular trafficking pathways critical for membrane protein presentation. Here, we presented only indirect evidence for the latter effect, demonstrating a reduction in two key proteins involved in these cardinal cellular processes. Future studies will focus on providing functional evidence for the changes in protein expression in rafts and membrane domains at the intact cellular-membrane level following miR-16 expression. Moreover, our current in vitro models cannot confirm that the observed changes in checkpoint protein expression affect melanoma immunogenicity; this will require validation in immune-competent animal models. Further elucidation of these regulatory mechanisms may offer novel strategies to enhance melanoma immunogenicity and improve responses to immune checkpoint blockade.

## Figures and Tables

**Table 1:** Primers for cloning the 3′UTR of SMPD1, VTI1b, CD80, or CD40 to a PsiCHECK vector.

Name	Sequence: (5′ to 3′)
psi_smpd1F	CCCGGGAATTCGTTTGGCCCCAGGGCCCACATTTG
psi_smpd1R	GGCCGCTCTAGGTTTTGGAGTCCAAGTCTCTTATC
psi_vti1bF	CCCGGGAATTCGTTTACTTCTATAGGGAAGGGTTT
psi_vti1bR	GGCCGCTCTAGGTTTGTGGCATACATTTTGCCAAC
psi_CD80F	CCCGGGAATTCGTTTCTAACTCTGGTGCTCTTTCC
psi_CD80R	GGCCGCTCTAGGTTTTGGTGTTAGACCTCTCTGCC
psi_CD40F	CCCGGGAATTCGTTTGGAACCCCAGGAGATCAATTTTCC
psi_CD40R	GGCCGCTCTAGGTTTACCCTTCTTCCCCACCCCAGACG
miR-16F	AGCTTGTCAGCAGTGCCTTAGCAGCACGTAAATATTGGCGTTAAGATTCTAAAATTATCT CCAGTATTAACTGTGCTGCTGAAGTAAGGTTGACG
miR-16R	AATTCGTCAACCTTACTTCAGCAGCACAGTTAATACTGGAGATAATTTTAGAATCTTAACG CCAATATTTACGTGCTGCTAAGGCACTGCTGACA

**Table 2:** Spearman rho correlation coefficients of the expression of checkpoint mRNAs.

PVRL2	TNSFSF18	TNSFF4	ICOSLG	CD274	CD40	LGALS9	CD80	PDCD1LG2	
0.24	0.47	0.51	0.61	0.73	0.74	0.79	0.90	0.90	CD86
0.17	0.49	0.49	0.52	0.83	0.67	0.73	0.89		PDCD1LG2
0.14	0.45	0.47	0.53	0.78	0.70	0.72			CD80
0.27	0.26	0.34	0.54	0.57	0.60				LGALS9
0.25	0.32	0.44	0.56	0.55					CD40
0.05	0.40	0.47	0.38						CD274
0.22	0.21	0.36							ICOSLG
0.01	0.31								TNSFF4
0.03									TNSFSF18

Red—Rho ≥ 0.7; Orange—0.5 ≤ Rho < 0.7; Yellow—0.2 ≤ Rho < 0.5; No color—Rho < 0.2.

**Table 3:** The mRNAs that are down-regulated >1.3-fold in melanoma cell lines over-expressing miR-16.

Gene Symbol	Fold Change— Over-Express miR-16 vs. Control	*p*-Value	Bioinformatics Possible Target of miR-16/15
VPS33B	−1.36448	0.0375063	√
WIPI2	−1.36762	0.0375063	√
MFAP3L	−1.37428	0.0654535	√
PPP1R11	−1.39183	0.0624488	√
TOMM34	−1.39962	0.0442763	√
TNPO3	−1.41463	0.0579612	
CDK4	−1.43391	0.0375063	√
RNF24	−1.43433	0.0375063	√
SLC15A4	−1.4514	0.0381192	√
TPRG1L	−1.47329	0.0375063	√
ACP2	−1.50057	0.0375063	√
TMEM109	−1.51314	0.0375063	√
VPS4A	−1.5148	0.0442763	√
AHCYL2	−1.55187	0.0375063	√
VTI1B	−1.56299	0.0375063	√
AGPAT3	−1.56375	0.0375063	√
MFSD12	−1.57871	0.0375063	
ICMT	−1.59596	0.0375063	√
SLC35A4	−1.65548	0.0546149	√
SLC20A2	−1.72137	0.0375063	√
ANAPC13	−1.74267	0.0466223	√
LMF2	−1.77151	0.0375063	√
YIF1B	−1.8387	0.0375063	√
ATG9A	−1.95334	0.0375063	√
RARS	−1.99813	0.0375063	
ARL2	−2.10958	0.0381192	√
ANKRD52	−2.16298	0.0577935	
SMPD1	−2.45929	0.0375063	√

**Table 4:** Gene ontologies that are over-represented among genes that are differentially under-expressed in melanoma cells expressing miR-16 (vs control melanoma cells), as determined using the PANTHER (Protein Analysis Through Evolutionary Relationships) Classification System [24,25].

Analysis Type:	PANTHER Overrepresentation Test (Released 11 July 2019)						
Annotation Version and Release Date:	GO Ontology Database; Released 3 July 2019						
Analyzed List:	Client Text Box Input (Homo sapiens)						
Reference List:	Homo sapiens (all genes in database)						
Test Type:	FISHER						
Correction:	FDR						
GO cellular component complete	Homo sapiens—REFLIST (20,996)	−41	(expected)	(over/under)	(-fold enrichment)	(raw *p*-value)	(FDR)
Late endosome membrane (GO:0031902)	136	4	0.27	+	15.06	1.57 × 10^−4^	4.00 × 10^−2^
Membrane-bounded organelle (GO:0043227)	12,535	36	24.48	+	1.47	1.61 × 10^−4^	3.28 × 10^−2^
Cytoplasmic part (GO:0044444)	9790	31	19.12	+	1.62	2.25 × 10^−4^	4.17 × 10^−2^
Endomembrane system (GO:0012505)	4494	22	8.78	+	2.51	6.18 × 10^−6^	4.20 × 10^−3^
Bounding membrane of organelle (GO:0098588)	2091	15	4.08	+	3.67	4.90 × 10^−6^	4.99 × 10^−3^
Organelle membrane (GO:0031090)	3525	20	6.88	+	2.91	2.31 × 10^−6^	4.70 × 10^−3^
Whole membrane (GO:0098805)	1683	12	3.29	+	3.65	6.25 × 10^−5^	2.55 × 10^−2^
Vacuolar membrane (GO:0005774)	417	6	0.81	+	7.37	1.58 × 10^−4^	3.58 × 10^−2^
Vacuolar part (GO:0044437)	575	7	1.12	+	6.23	1.19 × 10^−4^	3.46 × 10^−2^
Vacuole (GO:0005773)	789	9	1.54	+	5.84	1.83 × 10^−5^	9.31 × 10^−3^
Intracellular membrane-bounded organelle (GO:0043231)	11,046	33	21.57	+	1.53	2.62 × 10^−4^	4.44 × 10^−2^
Lysosome (GO:0005764)	684	7	1.34	+	5.24	3.40 × 10^−4^	5.33 × 10^−2^
Lytic vacuole (GO:0000323)	685	7	1.34	+	5.23	3.43 × 10^−4^	4.99 × 10^−2^
Secretory vesicle (GO:0099503)	995	9	1.94	+	4.63	1.09 × 10^−4^	3.70 × 10^−2^

## Data Availability

The data presented in this study are immediately available on request from the corresponding author (droravni@msn.com) due to technical issues (relating to the technical uploading of the data).

## Abbreviations

FDR	False Discovery Rate
IS	Immunological Synapse
MHC	Major Histocompatibility Complex
miRNA	micro-RNA
TCGA	Tumor Cancer Genome Atlas
TCR	T-Cell Receptor
